# Bladder metastasis with additional metastases in multiple other organs 4 years after radical nephrectomy for clear cell renal cell carcinoma: a case report and review of the literature 

**DOI:** 10.1186/s13256-022-03368-w

**Published:** 2022-04-03

**Authors:** Masayasu Urushibara, Masakazu Nagata, Taisuke Okumura, Hideto Kano, Yuuki Matsumoto, Shinichiro Tatsuoka, Tsunehiro Nenohi, Takeshi Shirakawa, Daisuke Kato, Masashi Kawamoto, Kazuhiro Ishizaka, Noriyuki Matsutani

**Affiliations:** 1 Department of Urology, Teikyo University Hospital, Mizonokuchi, 5-1-1, Futago, Takatsu-ku, Kawasaki, Kanagawa 213-8507 Japan; 2 Department of Diagnostic Pathology, Teikyo University Hospital, Mizonokuchi, 5-1-1, Futago, Takatsu-ku, Kawasaki, Kanagawa 213-8507 Japan; 3 Department of Thoracic Surgery, Teikyo University Hospital, Mizonokuchi, 5-1-1, Futago, Takatsu-ku, Kawasaki, Kanagawa 213-8507 Japan

**Keywords:** Renal cell carcinoma, Bladder metastasis, Immune checkpoint inhibitor, Postoperative prognostic factor, Interval to relapse

## Abstract

**Background:**

Renal cell carcinoma rarely metastasizes to the bladder, and its biological behavior is not yet fully understood.

**Case presentation:**

In our case (54-year-old Japanese woman), computed tomography evaluation suggested the presence of a bladder metastasis, associated with additional metastases in the lungs, mediastinal lymph nodes, ribs, and renal bed, 4 years after radical nephrectomy for renal cell carcinoma. The histological findings of the metastatic bladder tumor were consistent with those of clear cell carcinoma. The mediastinal lymph node, rib, and renal bed metastases responded to treatment with an immune checkpoint inhibitor administered for 12 months after surgery for the bladder and lung metastases. In patients with bladder metastasis, absence of metastases in other organs and an interval of more than 1 year after nephrectomy are known to be favorable prognostic factors. Interestingly, in our case, the bladder metastasis was detected more than 1 year after nephrectomy, which was a favorable factor, but there were also metastases in other organs, which was an unfavorable factor. Therefore, we reviewed the literature, including that pertaining to targeted therapy and immune checkpoint inhibitor therapy published in the last two decades, to analyze the clinical significance of the presence of additional metastasis in other organs in renal cell carcinoma (clear cell type, which is the predominant subtype) patients with bladder metastasis.

**Conclusions:**

Patients with bladder metastasis after nephrectomy for renal cell carcinoma also having metastases in other organs may respond to targeted therapy and immune checkpoint inhibitor therapy. This may suggest that the interval to relapse in the bladder after nephrectomy may be a more important prognostic factor than the presence of synchronous metastases in other organs.

## Introduction

Renal cell carcinomas (RCCs) have an unpredictable nature, making it very difficult to predict relapse after nephrectomy. Moreover, the clinical characteristics differ depending on the histological subtype. To the best of the authors’ knowledge, most previous studies on bladder metastasis from RCC have included various subtypes of RCC, that is, clear cell RCC, which is the predominant subtype, and the papillary, chromophobe, and sarcomatoid subtypes of RCC, because of the low incidence of this cancer. The development of targeted and immune checkpoint inhibitor (ICI) therapies has dramatically improved the cancer-specific survival (CSS) of metastatic RCC patients over the last two decades [1]. Herein, we report the case of a patient with metastases in multiple organs who was treated with an ICI after surgery for the bladder and lung metastases developing 4 years after radical nephrectomy for clear cell RCC. ICI therapy appears to yield longer CSS periods than targeted therapy. Furthermore, we reviewed the data of 41 cases of bladder metastasis from clear cell RCC published in the English- and Japanese-language literature from 2001 to 2021.

## Case report

**Fig. 1:**
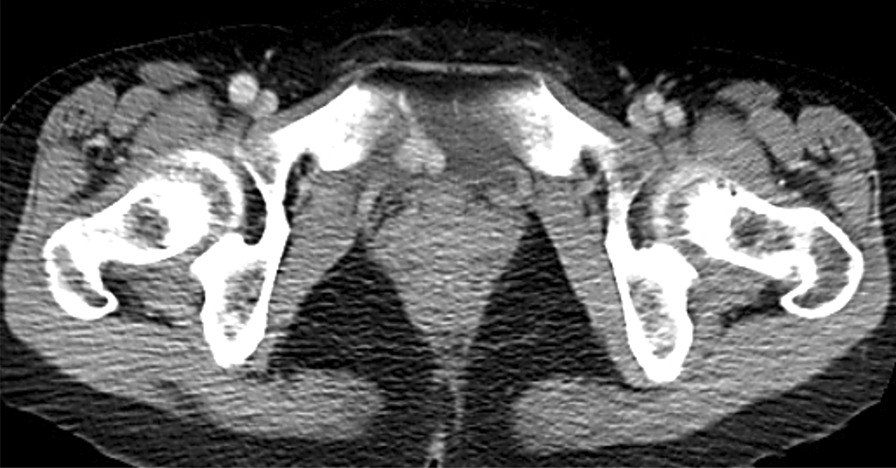
Contrast-enhanced pelvic computed tomographic image showing a well-enhanced intraluminal bladder mass, consisting of components

**Fig. 2:**
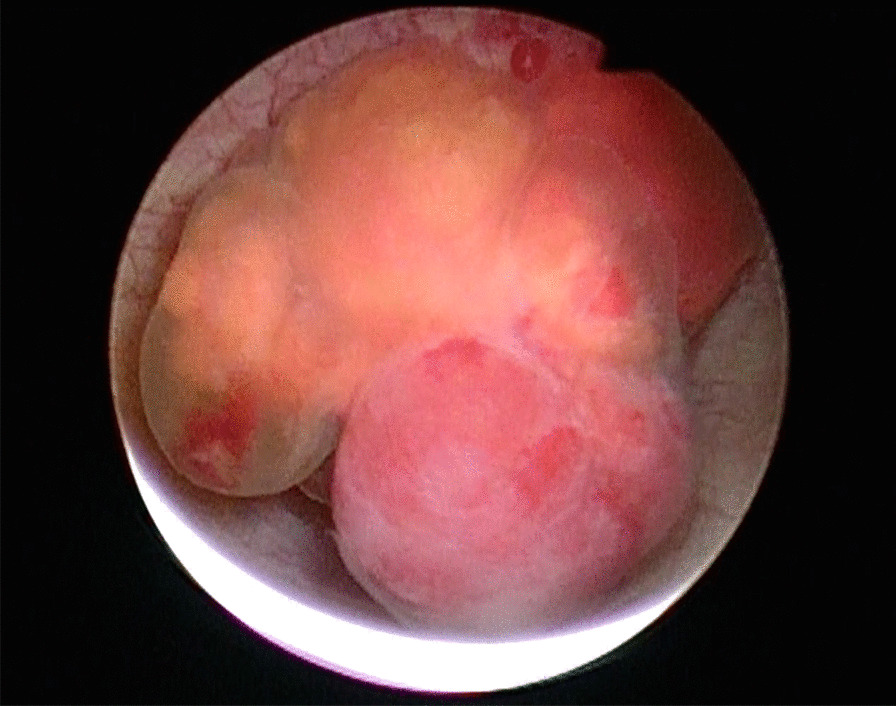
Cystoscopic findings. Spherical, red and yellow, nodular tumor surrounded by vessels

**Fig. 3:**
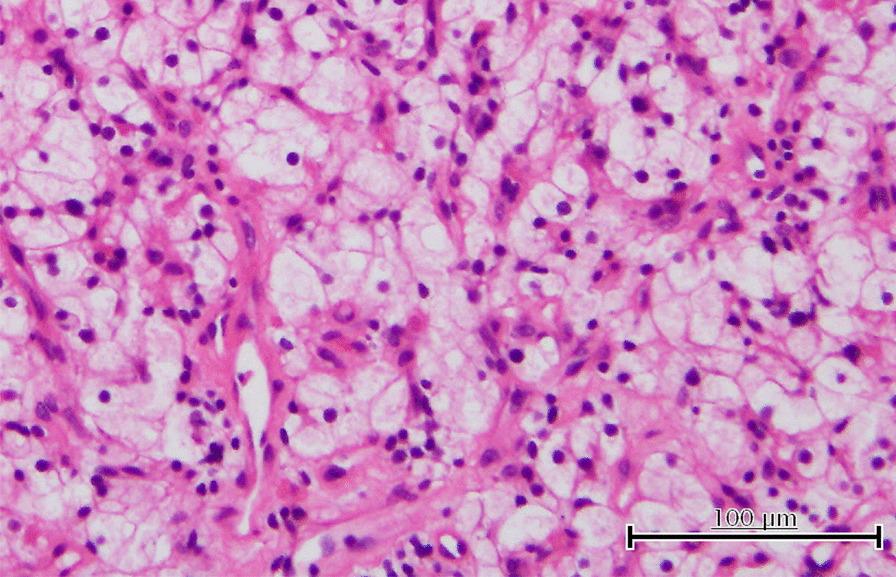
Histopathological findings. Clear cell carcinoma in the bladder. Hematoxylin–eosin stain, reduced from 40×

**Fig. 4:**
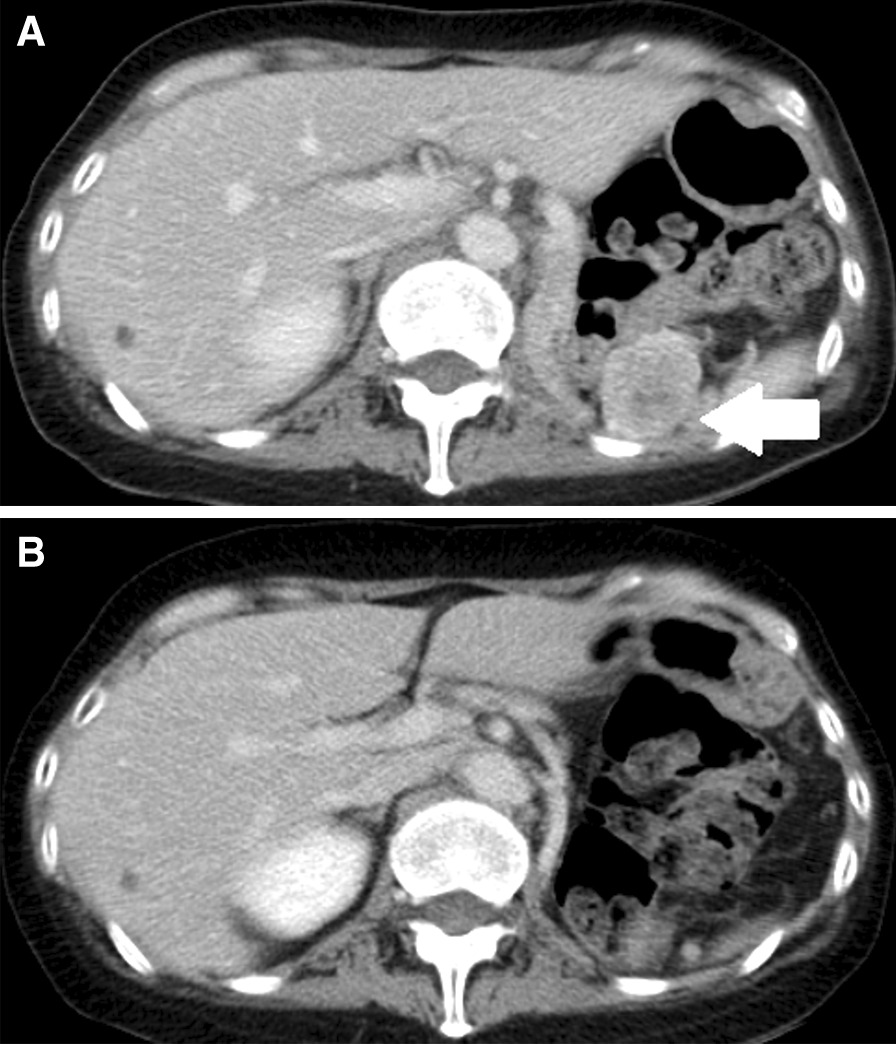
Change of CT findings of the recurrence in the left renal bed over time. **A** Pretreatment, 33 × 31 × 34 mm^3^ in size (white arrow). **B** After 12 months of immune checkpoint inhibitor therapy, the lesion size had reduced to 12 × 15 × 13 mm^3^

A 50-year-old Japanese woman, with no significant past medical history, was referred to our hospital with painless macroscopic hematuria. She was diagnosed as having a left-sided RCC, and treated by radical resection. Examination of the resected specimen showed a tumor measuring 100 mm in diameter with the features of clear cell RCC, with evidence of tumor invasion of the renal pelvis and renal vein, but no lymphatic invasion. Follow-up contrast-enhanced whole-body CT showed no relapse for 3 years after the surgery. However, 4 years after the surgery, contrast-enhanced CT revealed an enhancing lesion measuring about 20 mm in diameter in the bladder, along with metastases in the lungs, mediastinal lymph nodes, ribs, and renal bed (Fig. 1), although the patient had no symptoms. Cystoscopically, the single tumor consisted of several spherical components on the posterior bladder wall (Fig. 2). The bladder specimen was removed endoscopically, and histological examination revealed features consistent with clear cell RCC (WHO/ISUP nuclear grade 2), with no evidence of invasion of the muscular layer (Fig. 3). The patient fulfilled the IMDC risk criterion of neutrophilia, and was placed in the IMDC intermediate risk category. The bilateral pulmonary metastases were treated by endoscopic surgery (left basal segmentectomy by robotic-assisted thoracoscopic surgery after right lower lobe wedge resection by video-assisted thoracic surgery (VATS)), followed by ICI therapy (nivolumab and ipilimumab) for the mediastinal lymph node, rib (the patient refused surgery for the single rib metastasis), and renal bed metastases. The patient was started on ICI therapy, with each treatment cycle consisting of nivolumab (240 mg) and ipilimumab (1 mg/kg) administered concomitantly by intravenous injection once every 3 weeks. After three treatment cycles, the patient developed headache, fatigue, and slight fever and had red swollen eyelids. Laboratory examination revealed evidence of thyrotoxicosis and reduction of the plasma levels of adrenocorticotropic hormone and cortisol below the normal range, and the ICI therapy was suspended. The treatment was resumed soon after the start of steroid replacement therapy, and eventually, the recommended four cycles of nivolumab+ ipilimumab treatment were completed. Thereafter, nivolumab alone (480 mg, every 4 weeks) was administered, as scheduled, while the steroid replacement therapy was continued. After the eighth administration of nivolumab, thyroid hormone replacement was started to treat the hypothyroidism that had evolved gradually. However, by this time, the patient had already received 12 doses of nivolumab. By 12 months after the start of the ICI therapy, all the metastatic lesions had reduced to about one-third of their pretreatment size (Fig. 4).

## Discussion

We report the case of a patient with bladder metastasis, associated with synchronous metastases in multiple other organs, 4 years after nephrectomy for clear cell RCC. RCC can metastasize to many distant organs, such as lung, bone, skin, liver, and brain. On the other hand, RCC is rarely known to metastasize to the bladder, with bladder metastasis from RCC accounting for 1% of all secondary bladder tumors in 282 surgical and post mortem cases [2]. Matsumoto conducted a retrospective analysis of 65 cases of bladder metastasis from RCC reported in the English- and Japanese-language literature [3]. Among cases of bladder metastasis from RCC, the prevalence of cases with no additional metastases (62%) was higher than that of cases with additional synchronous metastases (38%); the additional metastatic organs were usually the lung, bone, and adrenal glands. Saitoh found that only 23 (2%) of 1451 autopsy cases of RCC had bladder metastasis, and of these, only 1 had no additional metastases in other organs [4]. Subclinical bladder metastasis is more likely to be detected in autopsy cases of RCC. It is not uncommon for relapse in cases of RCC to occur a long period after nephrectomy. Therefore, we propose defining “solitary bladder metastasis” as cases without additional metastasis at more than 2 years after surgery for bladder metastasis. To the best of the authors’ knowledge, only two cases of solitary bladder metastasis at the first visit or after nephrectomy in our review matched this definition. One patient with a solitary bladder metastasis at the first visit progressed to adrenal gland metastasis 24 months after the diagnosis of the bladder metastasis [5]. Another patient with a solitary bladder metastasis progressed to pancreatic metastasis 12 months later, and bone metastasis 13 months after nephrectomy [6]. Only 2 of the 32 patients in our review fulfilled the above criterion for solitary bladder metastasis, remaining free of additional metastases for 60 [7] and 72 [8] months, respectively, other than the cases who developed no additional metastases within 2 years after nephrectomy. Thus, different follow-up periods after the diagnosis of bladder metastasis may account for the different prevalences of solitary bladder metastasis from RCC in previously published reports.

The mechanism underlying the metastatic spread of RCC to the bladder remains unclear; however, four plausible pathways are suggested. The first is drop metastasis, or direct extension and implantation of tumor cells passing down the urinary tract into the bladder. Bladder metastasis near the ureteral orifice after nephrectomy for RCC as urothelial carcinoma or polypoid growth of the RCC undermining the urothelium and floating of tumor cells suggests such “drop metastasis” [9]. Second is direct hematogenous spread via the systemic circulation through invasion of the renal vein [10]. The third is a retrograde venous route, wherein tumor cell emboli travel from the renal vein into the numerous venous connections of the left renal vein [11]. Fourth is lymphatic spread, in which the cancer cells penetrate and embolize the lymphatic vessels and spread, facilitated by numerous interconnections between the lymphatic and vascular channels [12]. Babar reported RCC with metastasis to the bladder as well as distant organs, including bones, lungs, thyroid, contralateral renal vein, and inferior vena cava, 28 years after left radical nephrectomy and suggested that the hematogenous route was responsible for this metastasis [10]. There were eight cases of simultaneous bladder and other metastases at various times after nephrectomy in our review (median 54 months, range 1–336 months). The additional metastatic organs were the lungs in three cases, bone in three cases, liver in two cases, and thyroid, adrenal gland, renal-bed, ureter, and inferior vena cava in one case each (Table 1). In our case, it is hard to imagine that the bladder metastasis, which did not even reach the muscular layer, spread to additional organs by the hematogenous or lymphatic route.

**Table 1.** Treatment outcomes in clear cell renal cell carcinoma patients presenting with synchronous metastasis in other organs as well as bladder metastasis

Author	Year	Source of reference	Periods from nephrectomy (months)	Simultaneous additional metastasis	Therapy	Period from diagnosis of bladder metastasis (months)	Prognosis
Takahashi E	2001	[Article in Japanese] *Rinsho Hinyokika*	5	Bone	N.A.	7	Death from cancer
Kamota S	2003	[Article in Japanese] *Nihon Hinyokika Gakkai Zasshi*	4	Lung and ureter	–	22	Death from cancer
Kume H	2007	[Article in Japanese] *Nihon Hinyokika Gakkai Zasshi*	204	Bone	–	19	Death from another cause
Kume H	2007	[Article in Japanese] *Nihon Hinyokika Gakkai Zasshi*	48	Lung and liver	–	14	Death from cancer
Wang K	2015	*Oncol Lett*	60	Adrenal gland	Targeted	12	Alive with cancer
Li H	2016	*Oncol Lett*	1	Renal bed	–	0.5	Death from cancer
Groote RD	2018	*Ther Adv Urol*	60	Liver	Targeted	57	Alive with no cancer
Babar M	2019	*BMC Urol*	336	Lung, bone, thyroid, and inferior vena cava	Targeted→ICI	25	Alive with cancer
Present case	2022	Herein	48	Lung, bone, renal bed	ICI	15	Alive with cancer

Bladder metastasis with synchronous additional metastasis after nephrectomy in patients with clear cell renal cell carcinoma published in the literature over the last two decades. *Targeted* targeted therapy, *ICI* immune checkpoint inhibitor

Spread by implantation of tumor cells passing with the urinary stream into the bladder is unlikely to be accompanied by multiple metastases in other organs at the same time, 4 years after nephrectomy. Therefore, in our case, it is possible that the bladder metastasis itself was hematogenous in origin, with the tumor cells passing through the lung or spreading through the lymphatic route, via the mediastinal lymph nodes.

The purpose of endoscopic surgery for bladder metastasis is not only to allow histological examination of the tumor, but also tumor reduction. In our review, there was only one case of local recurrence of bladder metastasis (2%), in which a cancer-free state was achieved 36 months after a second surgery for bladder metastasis [8]. This suggests that bladder metastasis can only be controlled by surgery. Therefore, control of additional metastasis plays an important role in the prognosis after surgery for bladder metastasis.

The 2-year cancer-specific survival (CSS) rate after surgery for bladder metastasis was higher in the cases with solitary bladder metastasis than in those with additional metastases in other organs [3]. Furthermore, the 2-year CSS rate in patients who developed a bladder metastasis more than 1 year after nephrectomy was significantly higher than that in those who presented with a metastasis within 1 year of nephrectomy. Matsumoto reviewed the data of 35 cases (54%) reported from 1955 to 1999, among which only 3 (5%) received targeted therapy. In contrast, among the cases reviewed by us, ten patients had received targeted therapy (24%) and four patients (10%) had received ICI therapy. Three patients in our study developed bladder metastasis with synchronous metastasis in other organs within 6 months (at 1, 4, and 5 months) after nephrectomy. None of these three patients received either targeted or ICI therapy, due to poor performance status, anemia, and unstable glycemic control in one patient, and diagnosis before the era of these novel drug classes in the remaining two patients. All of the patients died of cancer, at 2 weeks, 7 months, and 22 months after diagnosis of bladder metastasis. Conversely, five patients developed bladder metastasis synchronously with other-organ metastasis more than 4 years (48, 60, 60, 204, and 336 months) after nephrectomy. Two patients, who received targeted therapy, were alive at 12 months (one case with cancer) [12] and 57 (one case, with no cancer) [13] months, one patient who received an ICI after targeted therapy was alive with cancer at 25 months [10], one died of RCC at 14 months, and one died of other cause at 19 months after the diagnosis of the bladder metastasis. These findings may suggest that the interval to relapse in the bladder after nephrectomy may be a more important prognostic factor than the presence of synchronous other-organ metastases. The overall objective response rates to targeted therapy were better in cases that developed later versus earlier after nephrectomy [14]. At least, in our review, targeted therapy or ICI therapy appears likely to have contributed to the survival by controlling the synchronous metastases in other organs developing in the longer term after nephrectomy.

Our review is certainly limited by its small size, retrospective design, adoption of different criteria for tumor staging, different treatments, and varying follow-up periods among the studies. Moreover, the follow-up period in our case, with synchronous other-organ metastases, was only 15 months. Nevertheless, to the best of the authors’ knowledge, this is the second report of synchronous other-site metastasis in a RCC patient with bladder metastasis, who received ICI therapy.

## Conclusion

The focus of this review is the clinical significance of additional metastases diagnosed in other organs synchronously with bladder metastasis in patients with clear cell RCC. The route of simultaneous spread to the bladder and other organs, as in our case reported here, is more likely to be systemic hematogenous or lymphatic spread simultaneously to the bladder and other organs rather than progression to other organs from the bladder metastasis. Bladder metastasis with synchronous metastases in other organs after nephrectomy for clear cell RCC, especially in the longer term, can be treated by targeted and/or ICI therapy, after surgery for the bladder metastasis performed not only to reduce the tumor volume but also allow histological diagnosis of the bladder metastasis.

## Data Availability

All data generated or analyzed during this study are included in this article and its supplementary information file.

## Abbreviations

RCC: Renal cell carcinoma
CT: Computed tomography
WHO: World Health Organization
ISUP: International Society of Urological Pathology
ICI: Immune checkpoint inhibitor
VATS: Video-assisted thoracic surgery
IMDC: International Metastatic RCC Database Consortium
CSS: Cancer-specific survival
